# Different effects of NK cells and NK-derived soluble factors on cell lines derived from primary or metastatic pancreatic cancers

**DOI:** 10.1007/s00262-022-03340-z

**Published:** 2022-11-30

**Authors:** Piera Filomena Fiore, Anna Laura Di Pace, Libenzio Adrian Conti, Nicola Tumino, Francesca Besi, Silvia Scaglione, Enrico Munari, Lorenzo Moretta, Paola Vacca

**Affiliations:** 1 Tumor Immunology Unit, Istituto di Ricovero e Cura a Carattere Scientifico (IRCCS) Bambino Gesù Children’s Hospital, Rome, Italy; 2 React4life SRL, 16121 Genoa, Italy; 3 Institute of Electronics, Information Engineering and Telecommunications (IEIIT), National Research Council of Italy, 16149 Genoa, Italy; 4 Department of Molecular and Translational Medicine, University of Brescia, Brescia, Italy; 5 Immunology Research Area, Innate Lymphoid Cells Unit, Bambino Gesù Children’s Hospital IRCCS, Rome, Italy; 6 Bambino Gesù Children’s Hospital IRCCS, Rome, Italy

**Keywords:** NK cells, Pancreatic adenocarcinoma, Exosomes, Soluble factors, 3D model

## Abstract

Natural killer (NK) cells are cytotoxic lymphoid cells that play a key role in defenses against tumors. However, their function may be severely impaired in patients with pancreatic adenocarcinoma (PA). Indeed, PA cells release soluble factors, thereby generating an immunosuppressive environment that dysregulates NK-cell cytolytic function and favors tumor immune evasion. Here, we analyzed the interactions between NK and PA cells using the PANC-1 and CAPAN-1 cell lines derived from a ductal PA and metastatic lesion, respectively. Metastatic and nonmetastatic cell lines were both able to impair NK cytolytic activity. An analysis of the effect of NK cells and NK-cell-derived exosomes revealed substantial differences between the two cell lines. Thus, NK cells displayed higher cytotoxicity against nonmetastatic PA cells than metastatic PA cells in both 2D cultures and in a 3D extracellular matrix cell system. In addition, NK-derived exosomes could penetrate only PANC-1 spheroids and induce cell killing. Remarkably, when PANC-1 cells were exposed to NK-derived soluble factors, they displayed substantial changes in the expression of genes involved in epithelial-to-mesenchymal transition (EMT) and acquired resistance to NK-mediated cytolysis. These results, together with their correlation with poor clinical outcomes in PA patients, suggest that the induction of resistance to cytolysis upon exposure to NK-derived soluble factors could reflect the occurrence of EMT in tumor cells. Our data indicate that a deeper investigation of the interaction between NK cells and tumor cells may be crucial for immunotherapy, possibly improving the outcome of PA treatment by targeting critical steps of NK-tumor cell crosstalk.

**Supplementary Information:**

The online version contains supplementary material available at 10.1007/s00262-022-03340-z.

## Introduction

Pancreatic adenocarcinoma (PA) is one of the most lethal tumors, as it has incidence and mortality rates [1, 2]. Surgical removal and adjuvant chemotherapy are currently the only therapeutic options for PA [3]. Unfortunately, the majority of patients have advanced metastatic disease at the time of diagnosis, and treatment is not effective. PA is characterized by a severe fibrotic response generating a dense stroma (desmoplasia), which may represent more than 80% of the tumor mass [4, 5]. The PA stroma is formed by different cellular components, including fibroblasts, inflammatory cells, pancreatic stellate cells, myofibroblasts, endothelial cells, and acellular components, such as collagens, hyaluronan and cytokines [6, 7]. All these components favor tumor progression, drug resistance and immune escape [4].

Although immune effector cells, such as natural killer (NK) cells, are potentially able to recognize and kill cancer cells, they may develop multiple mechanisms to evade the immune response. The ability to discriminate virus-infected or tumor cells from healthy “self” cells is primarily dependent on the loss of HLA class I molecules, which deliver inhibitory signals, and on the expression of activating stress molecules such as NKG2D and natural cytotoxic receptor (NCR) ligands [8]. NK-mediated cytotoxicity is regulated by a balance of signals delivered by activating and inhibitory receptors that may also lead to the release of cytotoxic granules containing perforin and granzymes [8, 9] and to cytokine production. Notably, NK exosomes also exert cytotoxic activity against tumor cells, representing an important tool of NK effector function [10]. Exosomes may represent promising therapeutic vehicles to transfer materials or drugs into target cells.

Several studies have shown that the expression of activating receptors such as NCRs, DNAM-1 and NKG2D is decreased in NK cells isolated from the peripheral blood (PB) of PA patients compared to NK cells from healthy donors (HD). Moreover, previous studies have demonstrated that both DNAM-1 and NKG2D receptors are involved in NK-mediated lysis of PA cells [11, 12].

In PA patients, NK cells show lower levels of cytotoxicity than NK cells from HD [13, 14]. The PA microenvironment promotes the expression of ligands of PD-1 inhibitory receptors such as PD-L1 and PD-L2 as well as the production of immunosuppressive molecules such as TGF-β, IL-10, and IDO-induced kynurenine. In addition, the PA stroma is infiltrated by myeloid-derived suppressor cells (MDSC) and tumor-associated macrophages (TAMs) [15]. Both MDSCs and TAMs play a critical role in impairing NK-cell function and compromising NK-based immunotherapy [16, 17].

NK cells represent an attractive tool for cancer immunotherapy due to their strong cytolytic activity and the possibility of infusing donor NK cells without the risk of GvHD; however, their role in PA is still unclear. Accordingly, studies investigating the interactions between pancreatic cancer cells and NK cells are needed to clarify both the potential cytolytic activity of NK cells against PA and the mechanism of resistance/escape from NK-mediated responses.

In this study, we analyzed the interactions between NK cells and two PA cell lines derived from primary or metastatic tumors. We show that both PA cell lines can inhibit NK effector function, compromising/altering different mechanisms involving NK receptors, primarily DNAM-1, NKG2D and PD-1. Importantly, metastatic PA cells are more resistant to NK-cell-mediated cytolytic activity in both 2D and 3D cell culture systems. Furthermore, we investigated the effects of soluble factors and exosomes produced by NK cells on PA cells. Our data show that NK-derived soluble factors may favor the selection of a subset of tumor cells acquiring resistance to NK cells as a result of the induction of epithelial to mesenchymal transition (EMT) of PA cells. On the other hand, NK-derived exosomes can induce cytotoxicity in PA cells and penetrate PA spheroids without inducing resistance to NK cells.

## Materials and methods

### Cell lines and cell culture

The NALM-18 (Childhood B acute lymphoblastic leukemia) cancer cell line was kindly provided by Dr Pende D. (IRCCS, Policlinico San Martino, Genoa, Italy) and cultured in RPMI 1640 (Euroclone, Milan, Italy) supplemented with 10% FBS (Euroclone), 1% penicillin/streptomycin (Euroclone) and 1% L-glutamine (Euroclone). PANC-1 (human pancreatic cancer cell; ATCC) cells were cultured in Dulbecco’s modified Eagle’s (DMEM) supplemented with 10% FBS, 1% penicillin/streptomycin and 1% L-glutamine. CAPAN-1 (human pancreatic cancer cell; ATCC) were cultured in RPMI supplemented with 20% FBS, 1% penicillin/streptomycin and 1% L-glutamine. All cell lines were cultured in a humidified atmosphere with 5% CO_2_ at 37 °C.

### NK-Cell isolation and expansion

NK cells were obtained from healthy volunteers provided by the blood transfusion center of Bambino Gesù Pediatric Hospital. The Ethical Committee of OPBG approved the study (825/2014) that was conducted in accordance with the ethical principles stated in the Declaration of Helsinki. Peripheral blood mononuclear cells (PBMCs) were separated by Ficoll-Hypaque density gradient centrifugation (Cederlane, Burlington, Ontario Canada). NK cells were isolated by RosetteSep human NK-cell enrichment cocktail (Stem Cell Technologies SARL, Grenoble, France). For expansion and activation, purified NK cells were seeded in 96-well U-bottom plates in MACS Medium® (Miltenyi Biotec) with IL2 (600 U/mL Novartis, Basilea, Switzerland). After two weeks of culture, activated NK cells did not express exhaustion and senescence markers and were used for cytotoxicity assays as previously described [18]. To obtain NK conditioned medium (NK-CM), activated NK cells were cultured for two weeks in RPMI 1640 supplemented with 10% FBS, 1% penicillin/streptomycin, and 1% L-glutamine for 48 h. In coculture experiments with PA cells, freshly isolated NK cells were added to PA cells in direct cell contact (PA cell to NK-cell ratio of 1:5) in RPMI supplemented with IL2 (600 U/mL).

### Cytotoxicity assay, degranulation and intracellular IFN-γ staining

PA cells or the NALM-18 cell line were used as NK-cell targets in the cytotoxicity assay. Target cells were labeled with Green Cell Tracker (CMFDA; Invitrogen, Thermo Fisher Scientific) according to the manufacturer’s instructions and then incubated with IL-2-activated NK cells for 4 h at different effector/target ratios. For experiments with blocking antibodies, anti-PD-L1 and anti-HLA-Cl I (A6136 clone Mouse IgM) mAbs were added at the start of NK incubation with the cell target. Propidium iodide (Sigma Aldrich, Saint Louis, MO, USA) was added to identify dead cells. Cells were acquired with a Beckman-Coulter Cytoflex-S flow cytometer, and live target cells were identified as CMFDA + PI − whereas dead target cells were identified as CMFDA + PI + . The percentage (%) of cell lysis was calculated as follows: % cell lysis = ((% of dead cells cultured with NK) − (% of spontaneous lysis))/(100 − (% of spontaneous lysis)) × 100.

NK-cell degranulation was determined by the cell surface expression of CD107a. NK cells were mixed with an equal number of NALM-18 cells for 4 h in the presence of monensin (BD Biosciences, GolgiStop) and APC-anti-CD107a (Miltenyi Biotec). At the end of stimulation, NK cells were stained with PC7-anti-CD56 (BD Biosciences), fixed and permeabilized with a Fixation and Permeabilization Kit (BD Biosciences) and incubated with intracellular PE-anti-IFNγ (Miltenyi Biotec).

### Monoclonal antibodies and cytofluorimetric analysis

For cytofluorimetric analysis, cells were stained with surface antibodies in PBS containing 5% FCS for 20 min at 4 °C. The following antibodies were used: IFNγ-PE, CD107a-APC, HLA-DR-PerCP, CD69-FITC, PD-1-APC (Miltenyi Biotec); CD56-PC7, NKp30-PE (Beckman Coulter); NKp46-V450, CD16 PerCP-Cy5,5, CD155-AF647, PD-L1 PE-CF59 (BD Biosciences); E-cadherin-APC (Thermo Fisher Scientific); N-cadherin-PE (Abcam); NKG2D-PE/Dazzle, DNAM1-PE, CD112-PerCP-Cy5,5; MICA/B PC7 (Biolegend); ULBP3 AF405, ULBP2/5/6 PE (R&D).

### Exosome isolation

As previously described [10], activated NK cells were seeded in exosome-free medium for 48 h in the presence of IL2. Then, the supernatants were collected and centrifuged at 300 × g for 5 min and at 2000 × g for 15 min. Following filtration, the supernatants were pelleted by high-speed centrifugation (100,000 × g for 70 min) (Optima MAX-XPN, Beckman, Brea CA, USA), and the exosomes were washed with phosphate-buffered saline (PBS). Isolated exosomes were resuspended in PBS and indirectly quantified by Bradford assay (Bio-Rad, Hercules, CA, USA).

### 3D tumor culture

3D cancer cell-laden hydrogels were prepared as previously described [19]. Briefly, alginate powder (Manugel GMB, FMC Biopolymer) was dissolved in Dulbecco’s Phosphate-Buffered Saline (DPBS) at a concentration of 1% w/v, and the solution was filtered through a 0.2-μm membrane under sterile conditions. PA cell lines were detached from plastic tissue culture flasks with 0.05% trypsin and resuspended at a density of 4 × 106 cells/mL in RPMI supplemented with 10% FBS, 1% penicillin/streptomycin and 1% L-glutamine. The PA cell suspension was mixed with the sterile alginate solution to obtain a final alginate concentration of 0.5% w/v and a cellular density of 2 × 106 cells/ml. The PA cell/alginate suspensions were dropped by a 21-gauge needle into a 0.5 M CaCl2 gelling bath at 37 °C to form alginate spheres. After washing the spheres with DI water to remove excess Ca, the PA cells/alginate spheres were cultured in 96 multiwells in 10% FBS, 1% penicillin/streptomycin, 1% L-glutamine and CaCl2 (5 mM) in a humidified environment (5% CO2) at 37 °C.

### 3D cytotoxicity assays and migration

NK-cell cytotoxicity in the 3D model was evaluated by adding NK cells to PA-GFP cell alginate sphere cultures. After 24 h of incubation, PA-alginate spheres were moved to 96-well imaging plates (Eppendorf), and then PI was added. After 30 min of PI incubation, cell viability was evaluated by fluorescence/confocal microscopy.

To evaluate NK-cell migration, PA cell alginate spheres were treated with alginate solubilizing solution (0.15 M NaCl and 55 mM sodium citrate, Sigma Aldrich) in a volume of 100 μl/alginate spheres, incubated at 37 °C until alginate fully dissolved (approximately 20 min), washed twice with complete medium and pelleted by centrifugation (1400 rpm/5 min). The cell suspension recovered from alginate hydrogels.

### Quantitative PCR

Total RNA extraction from PA cells was performed with Qiazol Lysis Reagent (Qiagen, Hilden, Germany) following the manufacturer’s protocol. The RNA was converted to cDNA by RT‒PCR. For RT‒PCR, the High-Capacity cDNA RT kit from Applied Biosystem (Foster City, CA, USA) was used. Real-time PCR was carried out with TaqMan™ Fast Advanced Master Mix (Applied Biosystems, Foster City, CA, USA). The following TaqMan™ Gene Expression assays were used in TaqMan® Array Human Epithelial to Mesenchymal Transition 96-well plates.

### Confocal microscopy

To evaluate the penetration of NK cells and NK-exosomes in PA spheroids, PA-GFP cells/alginate spheres were incubated for 24 h with NK cells stained with CM-DIL (Life Technologies) according to the manufacturer’s instructions or exosomes stained with PKH-67 dye (Merck, Darmstadt, Germany) according to [20]. After incubation, alginate spheres were moved to 96-well imaging plates, fixed in 4% paraformaldehyde (Sigma Aldrich, Saint Louis, MO, USA) for 30 min, washed with PBS and then incubated with Hoechst 33,342 (Sigma Aldrich) for 1 h at 4 °C. After a wash with PBS, the alginate spheres were covered with buffered glycerol solution.

Fluorescent signals were detected using an Olympus FV3000 confocal microscope equipped with FV315-SW version 2.1.4.198 software. The laser power, beam splitters, filter settings, pinhole diameters, and scan mode were the same for all examined samples of each staining. Fluorochrome unmixing was performed by the acquisition of an automated sequential collection of multichannel images to reduce spectral crosstalk between channels.

### Statistical analysis

The sample size used is indicated in the legend of each figure. Quantitative data are presented as the means ± SDs or means ± SEMs. Statistical analysis was performed with Prism 6 (GraphPad Software, San Diego, Calif). Normality was tested with the Shapiro‒Wilk test. The Mann‒Whitney test or Student's t test was used to determine statistical significance. Differences were considered significant at *p* < 0.05.

## Results

### Inhibitory effect of pancreatic adenocarcinoma cells on NK-cell function

**Fig. 1:**
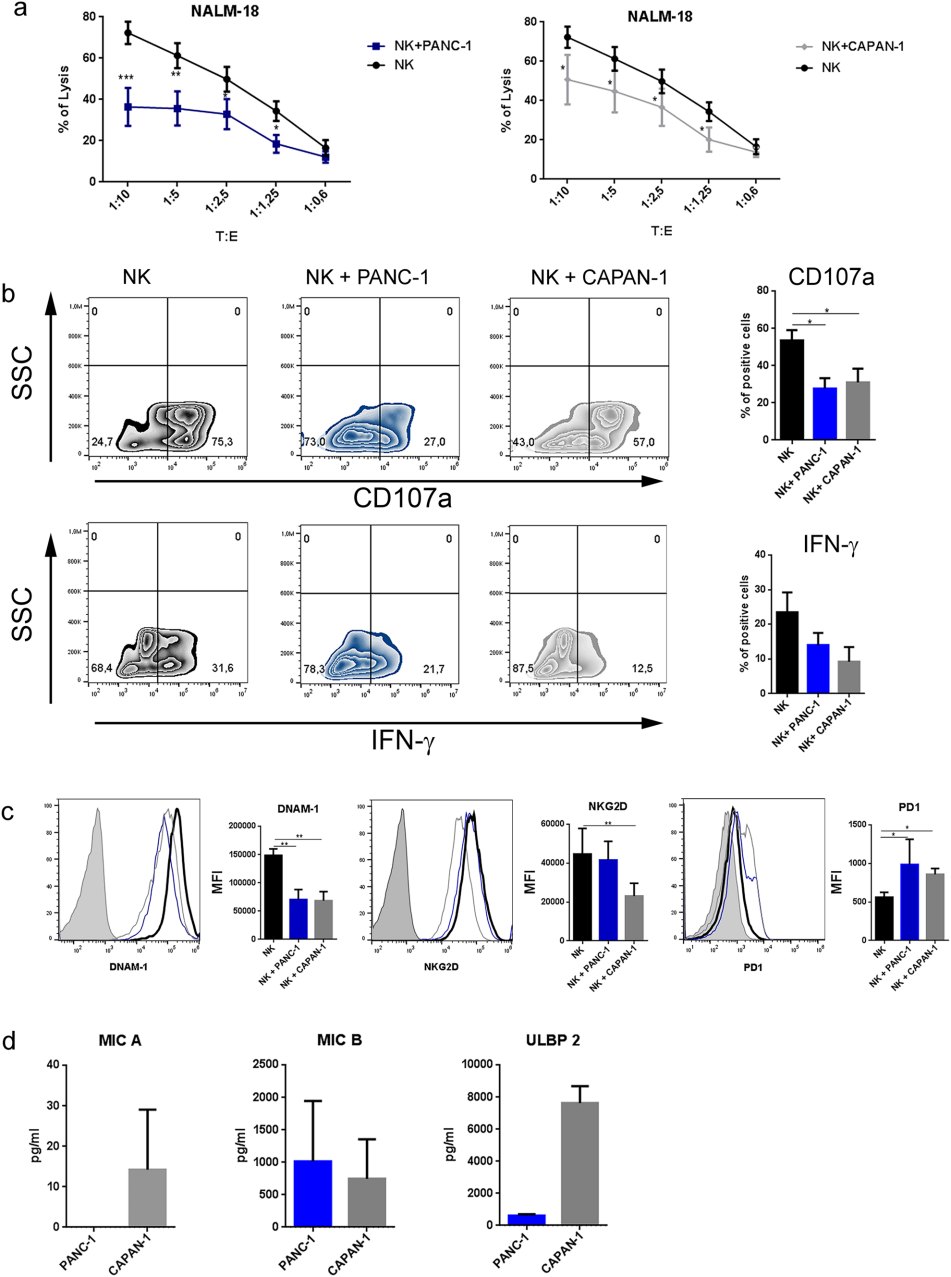
Impairment of NK-cell effector activity after direct contact with PA cell lines. NK cells were isolated from healthy donors and then cultured with IL2 in the presence or absence of PANC-1 or CAPAN-1 cells. After 6 days of culture, NK cells were isolated from the cocultures with PANC-1 or CAPAN-1 (indicated as NK + PANC-1 or NK + CAPAN-1, respectively) and functionally analyzed against NALM-18 target cells. **a** Cytotoxicity assay of NK cells isolated from PANC-1 or CAPAN-1 cocultures and NK cells cultured alone (NK) as a control at the indicated target:effector (T:E) ratios. Dead cells were evaluated as propidium iodide (PI)-positive cells by flow cytometry after 4 h of culture with target cells. Data indicate the average of 5 independent experiments ± SEM. Statistical significance was determined by paired Student's t test (**p < 0.01; ***p < 0.001). **b** Representative zebra plots of surface staining for CD107a (upper left panels) and intracellular staining for IFN-γ (lower left panels) on NK cells and NK + PANC-1 or NK + CAPAN-1 cells. Bars (right panels) represent the mean ± SEM of 4 different experiments. *p* values were calculated using paired Student's t test (*p < 0.05). **c** Surface expression of DNAM-1, NKG2D and PD-1 evaluated by flow cytometry. One representative experiment (left) out of 5 performed (black line control NK cells, blue line NK + PANC-1, gray line NK + CAPAN-1and filled gray line unstained NK cells). Bars (right) show the mean fluorescence intensity (MFI) of 5 different experiments for each cell line. *p* values were calculated using the Mann‒Whitney test (*p < 0.05, **p < 0.01). **d** Concentration (pg/ml) of MIC A, MIC B and ULBP 2 in PANC-1 and CAPAN-1 supernatants by ELISA

First, we analyzed the effect of pancreatic adenocarcinoma (PA) cell lines on NK-cell function. To assess whether PA could exert immunosuppressive activity, we used both the “nonmetastatic” and “metastatic” PA cell lines PANC-1 and CAPAN-1, respectively. These cells were cocultured with freshly isolated NK cells in the presence of IL2. Control NK cells containing IL2 alone were cultured in parallel. After 6 days, NK cells were isolated from cocultures, and their cytolytic activity was tested against the tumor cell line. As shown in Fig. 1a, NK cells cocultured with PA cells displayed an impairment of their cytolytic activity compared to control NK cells. Thus, both metastatic and nonmetastatic PA cell lines exert a similar immunosuppressive effect on NK-cell-mediated cytolysis. We then investigated whether PA cells could also inhibit NK-cell degranulation and cytokine production. Impaired degranulation (CD107a expression) and IFN-γ production were detected in NK cells cocultured with PA compared to control NK cells (Fig. 1b). Again, no substantial difference between metastatic and nonmetastatic cell lines was detected.

The impairment of NK-cell function, consequent to interaction with PA cells, could reflect the downregulation of the expression of major activating NK receptors that interact with their corresponding ligands that are frequently highly expressed on tumor cells. To this end, we analyzed the expression of activating receptors NCRs (NKp46, NKp30), DNAM-1 and NKG2D, the activation marker CD69, the triggering receptor CD16, and the checkpoint inhibitor PD-1 on NK cells under different culture conditions by flow cytometry. Coculture of NK cells with both PA tumor cells did not alter the expression of NKp46, NKp30, CD69, or CD16 (Supplementary Fig. 1a).

Notably, it has been shown that NK-cell-induced cytotoxicity of PA tumors is mainly mediated by NKG2D and DNAM-1 receptors [11, 12]. In line with these data, the analysis of the NK-cell receptor profile revealed a significant reduction in DNAM-1 on NK cells cocultured with PA cells (Fig. 1c). Notably, we found that NKG2D expression was downregulated in NK cells cultured with the metastatic cell line CAPAN-1 but not with the nonmetastatic PANC-1, suggesting differences in the inhibitory mechanisms (Fig. 1c). As previously reported, the proteolytic shedding of NKG2D ligands could be involved in tumor evasion from NK-mediated control [21]. Thus, we assessed whether NKG2D ligands (Supplementary Fig. 1b) were released from PA cells. Indeed, as shown in Fig. 1d, CAPAN-1 cells released high amounts of MIC-A and ULBP2, which are virtually absent in the PANC-1 supernatants. Notably, engagement of soluble NKG2D ligands can promote the endocytosis of NKG2D and its degradation by lysosomes [22, 23], revealing a possible mechanism of immune escape in the case of metastatic CAPAN-1. These results indicate that both metastatic and nonmetastatic PA cells are able to inhibit NK effector functions, possibly by using partially different NK receptor-related mechanisms. On the other hand, the expression of the immune inhibitory checkpoint PD-1 was increased in NK cells cultured with both PA cell lines, confirming the gain of a dysfunctional phenotype (Fig. 1c).

### Different susceptibilities of metastatic and nonmetastatic PA cells to lysis mediated by NK cells and NK-derived exosomes

**Fig. 2:**
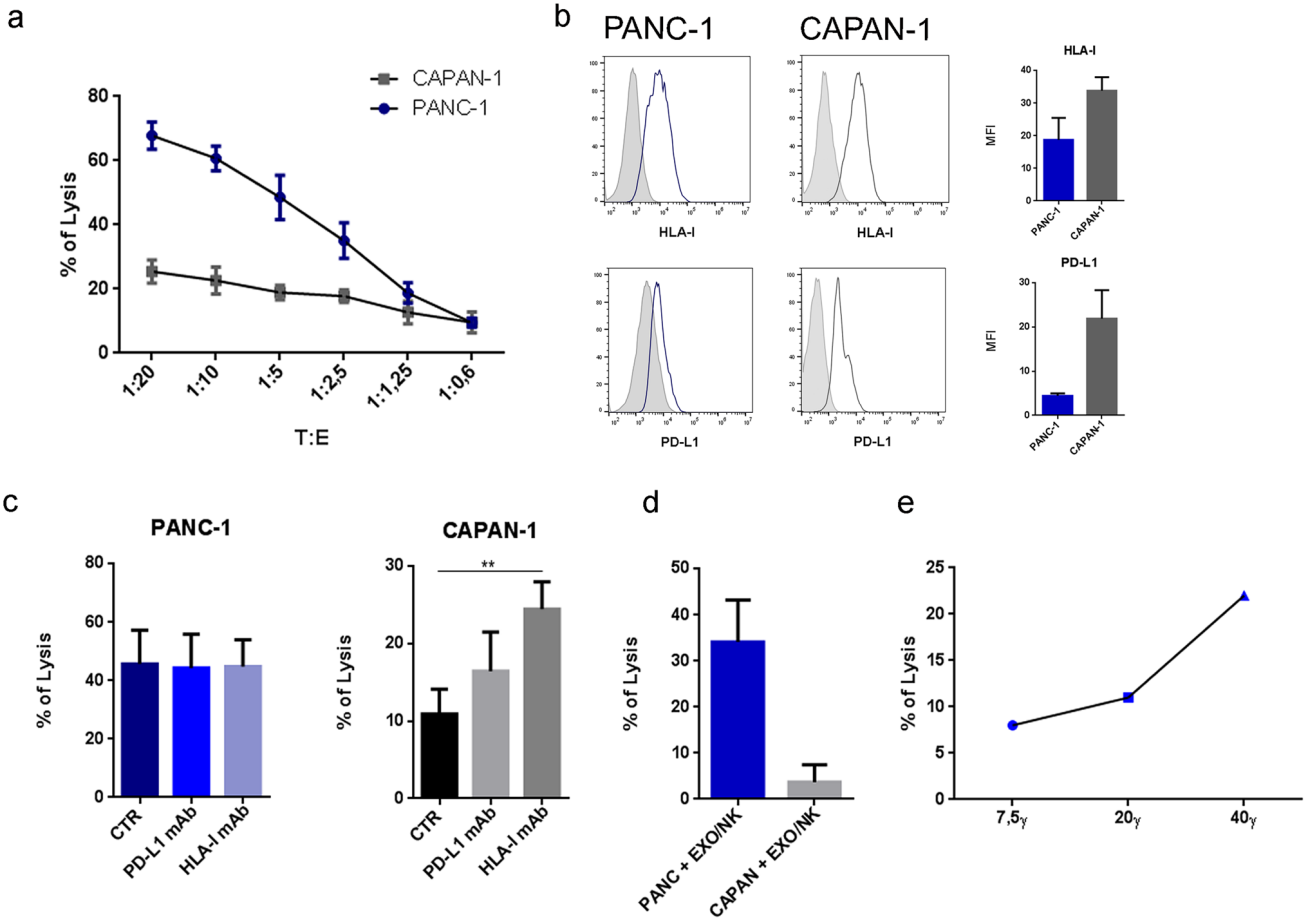
Role of HLA-I in metastatic and nonmetastatic PA cell sensitivity to NK-cell-mediated lysis. **a** Cytotoxicity of activated NK cells against PANC-1 and CAPAN-1 tumor cells after 4 h of incubation at the indicated T:E ratio. Dead cells were evaluated as PI-positive cells by flow cytometry. Data from 4 independent experiments for each cell line ± SEM. **b** Expression of HLA-I and PD-L1 on tumor cell lines. One representative experiment out of 5 was performed. Histograms show the means ± SEMs of the mean fluorescence intensity (MFI) of 5 different experiments for each cell line. **c** Role of PD-L1 and HLA-I in killing PA cell lines. NK cells were incubated with PA cells (1:10 T:E ratio) in the absence or in the presence of anti-PD-L1 and anti-HLA-1 blocking antibodies. The graph shows the killing efficiency expressed as a percentage of the killing. The cytotoxicity assay data shown were obtained from single PA cell cultures as targets and activated NK cells purified from five healthy donors after 4 h of coculture. Bar graphs are the mean ± SD. *p* values were calculated using a t test (*p < 0.05, **p < 0.01). **d** Cytotoxic assay on PANC-1 and CAPAN-1 target cells incubated with 40γ NK-derived exosomes. The percentage of PI^+^ cells was evaluated after 4 h of incubation with NK-exosomes. The mean ± SEM of five independent experiments is shown. **e** One representative experiment of PANC-1 cells incubated with different doses of NK-derived exosomes (7.5γ, 20γ, and 40γ). The percentages of dead cells (PI + cells) were evaluated after 4 h of incubation with NK-exosomes

We next investigated whether the PA cell lines were susceptible to NK-cell-mediated lysis. In line with previous studies, we found that metastatic CAPAN-1 cells are more resistant to NK-cell cytolytic activity than nonmetastatic PANC-1 cells (Fig. 2a). Similar results were observed using other metastatic and nonmetastatic PA cell lines (Supplementary Fig. 2). To clarify the mechanism(s) underlying these differences, we investigated the expression of the ligands of the main inhibitory NK receptors on the surface of PA cells. HLA-I and PD-L1 molecules are two important ligands that may confer tumor cell resistance to NK-cell-mediated killing, thus favoring immune escape. Thus, we analyzed the expression of HLA-I and PD-L1 in metastatic and nonmetastatic PA cell lines. As shown in Fig. 2b, CAPAN-1 cells expressed higher levels of HLA-I and PD-L1 molecules than PANC-1 cells. The high expression of HLA-I and PD-L1 molecules on metastatic PA cells could contribute explaining their higher resistance to NK-cell-mediated lysis. To assess this possibility, we analyzed the effect of blocking PD-L1 and HLA-I with mAbs disrupting the interaction between the inhibitory receptors on NK cells and their ligands on tumor cells. As shown in Fig. 2c, HLA-I blockade resulted in an increase in NK-mediated cytolysis of CAPAN-1 cells but not PANC-1 cells, indicating a different role of HLA-I in the resistance of PANC-1 and CAPAN-1 cells. Notably, PD-L1 blockade did not change the sensitivity to NK-cell-mediated lysis in either PA cell line. These data reflect the low or absent expression of PD-1 on the surface of NK cells isolated from healthy donors.

NK cells can efficiently release soluble factors and exosomes with cytotoxic potential. In this context, we have previously shown that NK-cell-derived exosomes (EXOs) can efficiently penetrate into and kill tumor cells [10]. Thus, purified exosomes derived from NK cells were tested for their cytotoxic activity against PA cell lines. Notably, regarding NK-cell-mediated killing (Fig. 2a), CAPAN-1 cells were more resistant than PANC-1 cells to the cytolytic activity of NK-derived exosomes (Fig. 2d). Since the PANC-1-cell line was more sensitive to NK-derived exosome-mediated lysis, we used this cell line to perform the cytotoxic assay at different exosome concentrations. As shown in Fig. 2e, the cytotoxic effect is clearly dose dependent.

Altogether, these data (Figs. 1 and 2) clearly indicate that metastatic PA cells, unlike nonmetastatic PA cells, exploit several mechanisms to elude the antitumor activity of NK cells.

### The interaction of nonmetastatic PA cells with NK cells can induce resistance to cytolysis

**Fig. 3:**
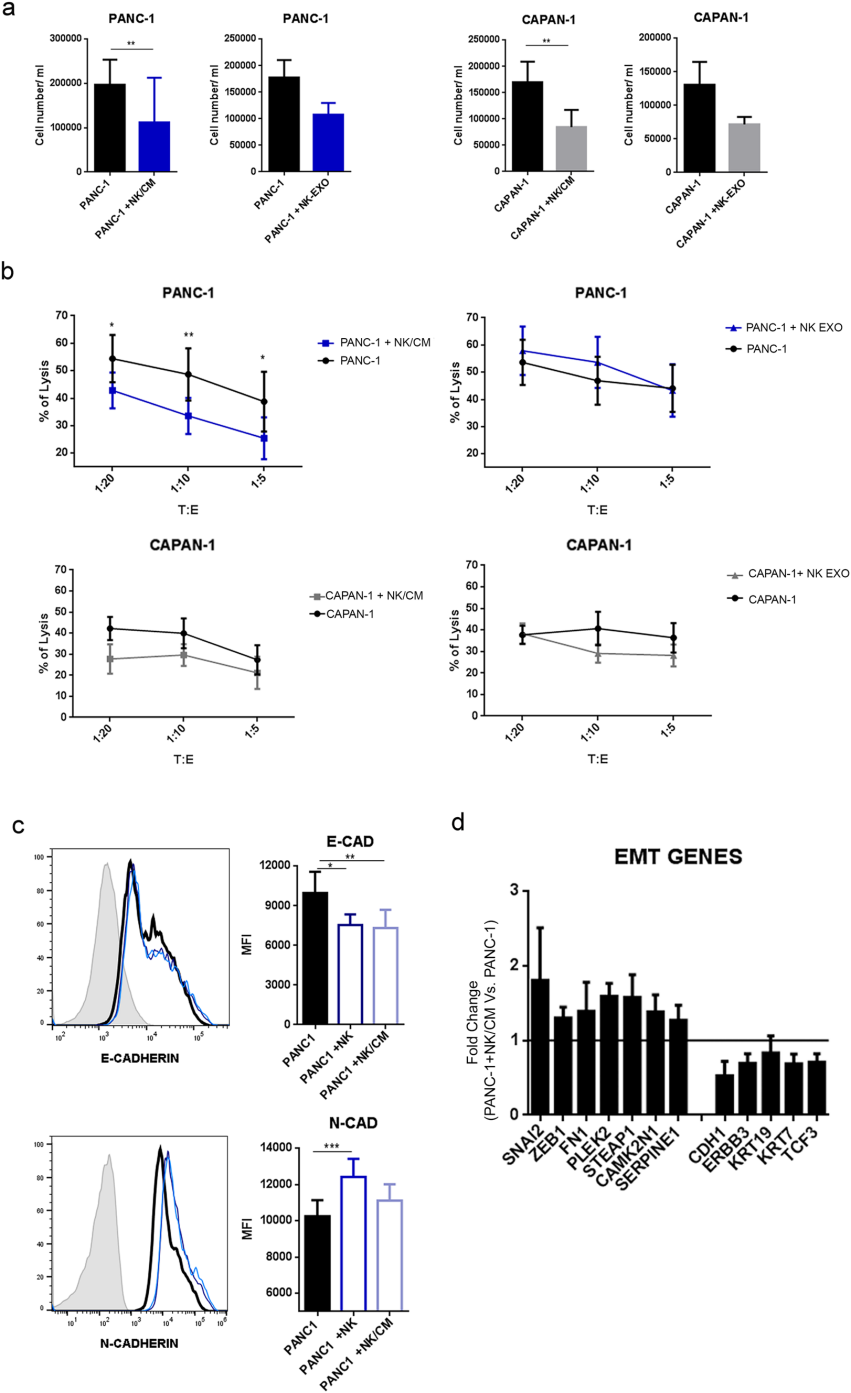
NK-derived soluble factors induce EMT in PA cells, reflecting increased tumor resistance. **a** PA cell number after 24 h of incubation with NK/CM. Data are the mean of 4 independent experiments for each cell line ± SEM (*p < 0.05; **p < 0.01). **b** Cytotoxicity of activated NK cells against PA cells pretreated or not with NK conditioned medium (NK/CM) for 24 h. NK cells were incubated with PA cells for 4 h at the indicated T:E ratios, and target cell viability was evaluated by flow cytometry (as PI^+^ cells). Data shown are the average of 6 independent experiments ± SEM. Statistical significance was determined by paired Student's t test (*p < 0.05; **p < 0.01). **c** E-CAD and N-CAD expression was measured by flow cytometry in conditioned PA cells and untreated PA cells. Histograms (left panel) and bars (right panel) of mean fluorescence intensity (MFI) ± SEM of 6 different experiments. Statistical significance was determined by paired Student's t test (*p < 0.05; **p < 0.01) **d** mRNA fold change of mesenchymal markers (SNAI2, ZEB1, FN1, PLEK2, STEAP1, CAMK2N1 and SERPINE1) and epithelial markers (CDH1, ERBB3, KRT19, KRT7 and TCF3) of PANC-1 cultured (24 h) with NK/CM versus PANC-1 cultured alone. The results are the means ± SEMs of different experiments using NK/CM derived from 3 different NK donors

Despite some promising results obtained in vitro, the response to immunotherapy of PA patients is largely ineffective, possibly involving, at least in part, an impairment of NK-cell function. Thus, we further investigated whether NK-derived soluble factors or could affect the sensitivity to NK-mediated killing. To this end, PA cells were cultured in the absence or in the presence of NK-derived exosomes (EXO-NK) or NK-cell-derived soluble factors, referred to as “conditioned medium (CM/NK)”. After 24 h of treatment with EXO- or the number of both PA cell types decreased (Fig. 3a), indicating that both EXO and could mediate a partial antitumor effect. Subsequently, to further investigate the effects of EXO and on PA cells, we assessed the sensitivity to NK-mediated lysis of PA cells leftover after coculture. The PA cells residual upon exposure to EXO-NK displayed a similar susceptibility to NK-mediated lysis compared to PA cells that were untreated with EXO-NK. In contrast, PA cells exposed to CM/NK showed different susceptibility to lysis compared to untreated tumor cells. In particular, only nonmetastatic PANC-1 cells left after exposure to CM/NK cells acquired significant resistance to NK cells (Fig. 3b). Taken together, these results indicate that NK-derived soluble factors can mediate cytotoxicity against PA cells but that may also favor the selection of a subset of tumor cells that have acquired more resistance to NK-cell lysis.

**Fig. 4.**
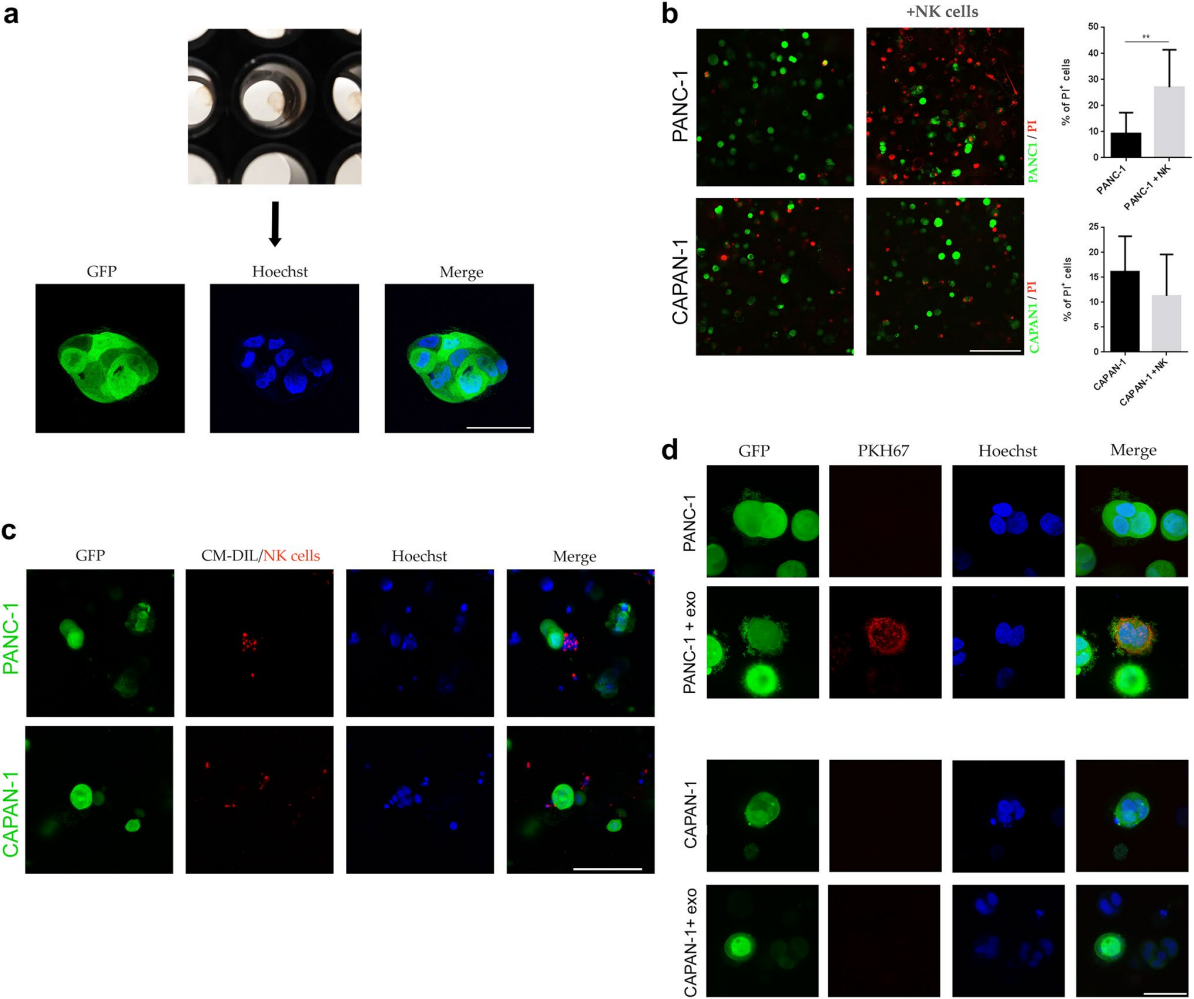
3D PA cells cultured in the absence or presence of NK cells or NK-derived exosomes. **a** Representative image of GFP-PA cell spheroids after 5 days of culture. Nuclei were stained with Hoechst (bar = 50 μm). **b** Necrotic spheroids after 24 h of coculture with NK cells. Dead cells were stained with PI. Histograms show the means ± SEMs of PI^+^ cells from 3 different experiments (bar = 200 μm). **c** NK-cell migration in GFP-PANC-1 and GFP-CAPAN-1 alginate spheres after 24 h of coculture. NK cells were labeled in red with CM-DIL (bar = 100 μm). **d** NK-exosome uptake in GFP-PANC-1 and GFP-CAPAN-1 spheroids. Cells were incubated with NK-exosomes labeled with PKH67 (red), and after 24 h, their internalization was evaluated (bar = 40 μm)

Epithelial to mesenchymal transition (EMT) represents one of the most important mechanisms by which tumor cells acquire drug and/or radio resistance and increase their metastatic potential. Thus, in some instances, NK cells can even favor the aggressiveness of tumor cells by inducing phenotypic changes revealing EMT [24, 25]. We investigated whether resistance to lysis that PANC-1 cells acquired upon conditioning with NK-derived soluble factors could be related, at least in part, to the phenotypic switch of tumor cells. To this end, PANC-1 cells were cultured in the presence or in the absence of NK cells or NK/CM. After 24 h of coculture, flow cytometric analysis showed that conditioned PANC-1 cells downregulated E-cadherin and upregulated N-cadherin (Fig. 4c). In view of these results, we further investigated this possibility. RT‒PCR analysis of the expression of genes involved in EMT. As shown in Fig. 3d, upon exposure to NK/CM, PANC-1 cells acquired a gene profile typical of EMT, as revealed by the concomitant upregulation of genes promoting the mesenchymal phenotype (SNAI2, ZEB1, FN1, PLEK2, STEAP1, CAMK2N1 and SERPINE1) and the downregulation of genes promoting the epithelial phenotype (CDH1, ERBB3, KRT19, KRT7 and TCF3). These data suggest that soluble factors released by NK cells can induce nonmetastatic PA cells to acquire resistance to NK-mediated killing, possibly reflecting the occurrence of EMT. Furthermore, we also found that NK/CM induced increased expression of IDO. IDO-induced kynurenine is known to be a crucial player in the immunosuppressive tumor microenvironment (Supplementary Fig. 3). To further validate our data and verify the possible correlation between modulated EMT genes and tumor aggressiveness in conditioned PANC-1 cells, we queried the R2 pancreatic tumor gene expression dataset. We found a correlation between the upregulation of CDH2, SLUG, STEAP1 and Serpine1, the downregulation of CHD1 and ERBB3 and poor clinical outcome in PA patients (Supplementary Fig. 4).

### NK-cell migration and cytotoxic activity in a 3D pancreatic tumor model

3D in vitro cancer models are an important tool to mimic, at least in part, the in vivo tumor structure. 3D PA cultures were generated using alginate hydrogel. Upon growth in alginate spheres, the PA cell lines could generate spheroid structures closer than 2D cultures to the in vivo tumor organization, as shown in Fig. 4a. First, we analyzed whether NK cells had any effect on PA 3D cultures. To this end, NK cells were added to PA spheroids, and the viability of tumor cells was evaluated after 24 h. As shown in Fig. 4b, NK cells substantially damaged PANC-1 spheroids, while CAPAN-1 spheroids were not affected. These data are in agreement with those of the 2D cultures represented in Fig. 2a. To understand whether differences in PA cell susceptibility to NK cells could be related to differences in NK-cell infiltration. 3D GFP-PA cells were cultured with CM-DIL-labeled NK cells for 24 h, and NK infiltration in the spheroids was assessed by confocal microscopy (Supplementary Fig. 5). NK cells permeated the alginate spheres and colocalized with PANC-1 spheroids but not with CAPAN-1 spheroids (Fig. 4c). In addition, the number of infiltrating NK cells was higher in PANC-1 spheroids than in CAPAN-1 spheroids (Supplementary Fig. 6a). We also investigated whether the invasion of NK cells could modulate the surface expression of HLA-I and PD-L1 in tumor spheroids. As shown in Supplementary Fig. 6b, we could not detect a significant increase in these markers in the presence of NK-cell infiltration. Taken together, these results revealed another possible mechanism by which metastatic PA cells may evade NK-cell killing, namely, interference with immune effector cell invasion.

We next used the same 3D assays to test the ability of NK exosomes to penetrate PA spheroids. Figure 4d shows that NK exosomes are internalized and accumulate in PANC-1 but not CAPAN-1 spheroids. These results indicate that metastatic and nonmetastatic PA spheroids are differently infiltrated by NK cells and display substantial differences in their susceptibility to NK-mediated killing by mechanisms based both on cell-to-cell contact and on soluble factors.

## Discussion

In the present study, we provide new information on the interactions occurring between NK cells and pancreatic adenocarcinomas (). In particular, we show that both the PANC-1-cell line derived from ductal PA and the CAPAN-1-cell line derived from a PA metastatic lesion are able to strongly inhibit the antitumor activity of NK cells. On the other hand, NK cells displayed a higher cytotoxicity against nonmetastatic PA cells than metastatic PA cells in both 2D cultures and in a 3D extracellular matrix cell system. Remarkably, nonmetastatic (PANC-1) cells, upon exposure to NK soluble factors, may acquire resistance to NK-mediated cytolysis. This acquired resistance is likely to reflect EMT, which is characterized by the concomitant upregulation of mesenchymal surface markers and genes and downregulation of epithelial markers and genes, suggesting a possible paradoxical effect of NK cells in favoring tumor progression, at least under certain conditions, as previously suggested for melanoma cell lines [24].

Pancreatic adenocarcinoma (PA) is one of the most lethal tumors with a high rate of incidence and mortality and a poor response to current therapies [1, 2]. Although the hypoxic, fibrotic and immunosuppressive PA environment impairs the recruitment and cytotoxic function of antitumor effector cells, previous studies revealed a positive correlation between NK-cell numbers and recurrence-free survival of PA patients [26]. This suggests that NK cells could represent a possible tool in PA treatment. In line with previous studies, we found that NK cells were able to kill both metastatic and nonmetastatic PA tumor cells, although with different efficiencies. In particular, PANC-1 cells showed a higher susceptibility to NK-mediated lysis than CAPAN-1 cells. Moreover, HLA-Cl I masking experiments revealed that the different susceptibility to NK-mediated killing is related, at least in part, to different levels of HLA-Cl I surface expression (higher in CAPAN-1 cells than in PANC-1 cells). We further analyzed the ability of NK cells to lyse PA cells in a three-dimensional (3D) culture system. 3D cultures represent a relevant and more informative preclinical model than 2D monolayers, as they can better mimic the structural and biological complexity of solid tumors. PA 3D cultures were generated using alginate hydrogel, where both CAPAN-1 and PANC-1 cell lines can form spheroid structures. PA 3D cultures confirmed the higher susceptibility of PANC-1 cells to NK-mediated lysis than CAPAN-1 cells. These data suggest that the 3D cell organization does not compromise the NK killing ability. These experiments also revealed that different PA cell lines display different chemoattractive behaviors toward NK cells. Thus, in the alginate hydrogel, NK cells migrate to PANC-1 spheroids and interact with them but not with CAPAN-1 spheroids. Not surprisingly, this defective localization of NK cells in CAPAN-1 spheroids further compromised their antitumor activity that, instead, was evident in PANC-1 spheroids.

Cancer cells can shape the tumor microenvironment through the release of soluble factors and by recruiting or altering the function of other cell populations, such as regulatory T cells (Treg), myeloid-derived suppressor cells (MDSC) and alternative macrophages (M2), which may affect the antitumor activity of NK cells. In this context, we provide evidence that both PANC-1 and CAPAN-1-cell lines inhibit the antitumor activity of NK cells. As previously shown, the activating receptors DNAM-1 and NKG2D play a major role in the NK-mediated lysis of PA cells. We found that the surface expression of DNAM-1 in NK cells was reduced upon coculture with both PANC-1 and CAPAN-1 cells. On the other hand, only the CAPAN-1 cell line was able to induce downregulation of NKG2D. This may be due to shedding of NKG2D ligands expressed on tumor cells, which, in turn, favors the internalization of NKG2D in NK cells. Indeed, CAPAN-1 cells, but not PANC-1 cells, release high amounts of soluble MICA and ULBP2 in the culture medium. Consistent with these data in vitro, in patients with metastatic PA, there are high serum concentrations of soluble NKG2D ligands and low percentages of NKG2D^+^ cells [27, 28].

NK cells have been shown to release soluble factors and microvesicles that may exert antitumor activity. Based on these findings, we investigated the effect of NK soluble factors on PA cells. Interestingly, after incubation with NK-derived soluble factors, PANC-1 cells (more susceptible to NK-cell-mediated killing) significantly increased their resistance to lysis. These paradoxical data suggest that tumor cells susceptible to NK cytotoxic activity could become resistant in an environment conditioned by soluble factors released by NK. In contrast, CAPAN-1 did not significantly increase their resistance to lysis. This is likely to reflect their high starting levels of resistance to NK-mediated cytotoxicity. Importantly, in PANC-1 cells exposed to NK-cell supernatants, we also detected downregulation of E-cadherin expression and upregulation of N-cadherin, suggesting the occurrence of EMT that may participate in the acquisition of resistance to NK-mediated killing. This concept is further supported by the concomitant upregulation of genes related to the mesenchymal phenotype and the downregulation of genes promoting the epithelial phenotype.

In addition to soluble factors, NK cells can also release exosomes, which function not only as cargos but also as cytolytic tools against tumors. Indeed, we show that EXO-NK are able to induce lysis of PA cells. Notably, CAPAN-1 cells were more resistant to both NK-mediated and EXO-mediated lysis than PANC-1 cells. Interestingly, unlike soluble factors present in NK-conditioned medium, EXO-NK did not induce PA cells to acquire resistance to NK-mediated lysis. EXO-NK cells could be a valuable instrument to improve NK-cell-based therapies. Thus, their ability to mediate a cytotoxic effect against tumor cells may complement the antitumor activity of NK cells. In this context, in PA 3D cultures, we show that EXO- are able to penetrate PA spheroids. In addition, EXO-NK are more efficiently internalized within PANC-1 cells than CAPAN-1 spheroids, in line with differences detected in the EXO-NK killing experiment performed in 2D cultures. Since 3D models are more similar to the in vivo organization of the tumor and TME, our present results may improve our knowledge of the mechanisms by which metastatic PA tumor cells may escape the immune response.

In conclusion, our data show that not only NK cells but also soluble factors and exosomes released by NK cells may affect PA cells. These data could be crucial in optimizing NK-cell-based immunotherapy.

## Supplementary Information

Below is the link to the electronic supplementary material.

Supplementary file1 (DOCX 2444 KB)
