# Supplementary material for: Different effects of NK cells and NK-derived soluble factors on cell lines derived from primary or metastatic pancreatic cancers
Source: Cancer Immunol Immunother. 2022 Nov 30;72(6):1417–28. doi: 10.1007/s00262-022-03340-z (PMC10198856; doi:10.1007/s00262-022-03340-z)
Supplement: Supplementary file 1 — Supplementary file1 (DOCX 2444 KB) [file 262_2022_3340_MOESM1_ESM.docx]

Supplementary Material


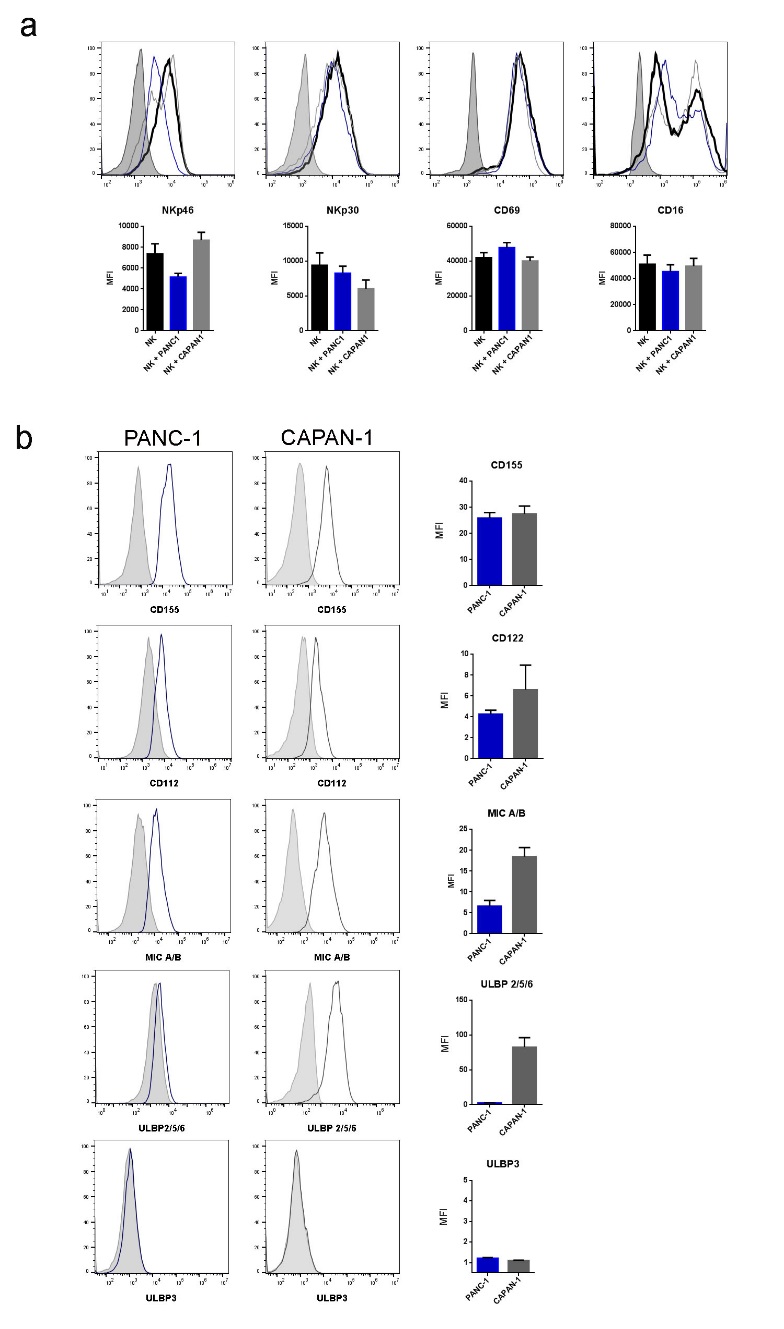


**Figure S1.** (**a**) NK cells were isolated from healthy donors and then cultured with IL2 in the presence or in the absence of PANC-1 or CAPAN-1 cells. After 6 days of culture, NK cells were isolated from the co-cultures with PANC-1 or CAPAN-1 (indicated as NK+PANC-1 or NK+CAPAN-1, respectively) and surface expression of NKp46, NKp30, CD69, and CD16 were evaluated by flow–cytometry. In the upper panel one representative experiment (black line control NK cells, blue line NK+PANC-1, grey line NK+CAPAN-1 and filled gray line unstained NK cells) out of 5 performed . Bars (lower panel) show the mean fluorescence intensity (MFI) ±SEM of 5 different experiments for each cell lines. (b) Expression of CD155, CD122, MIC A/B, ULBP 2/5/6 and ULBP3 on tumor cell lines. One representative experiment out of 5 performed (left panels). Bars (right panel) show the MFI±SEM of 5 different experiments for each cell lines.


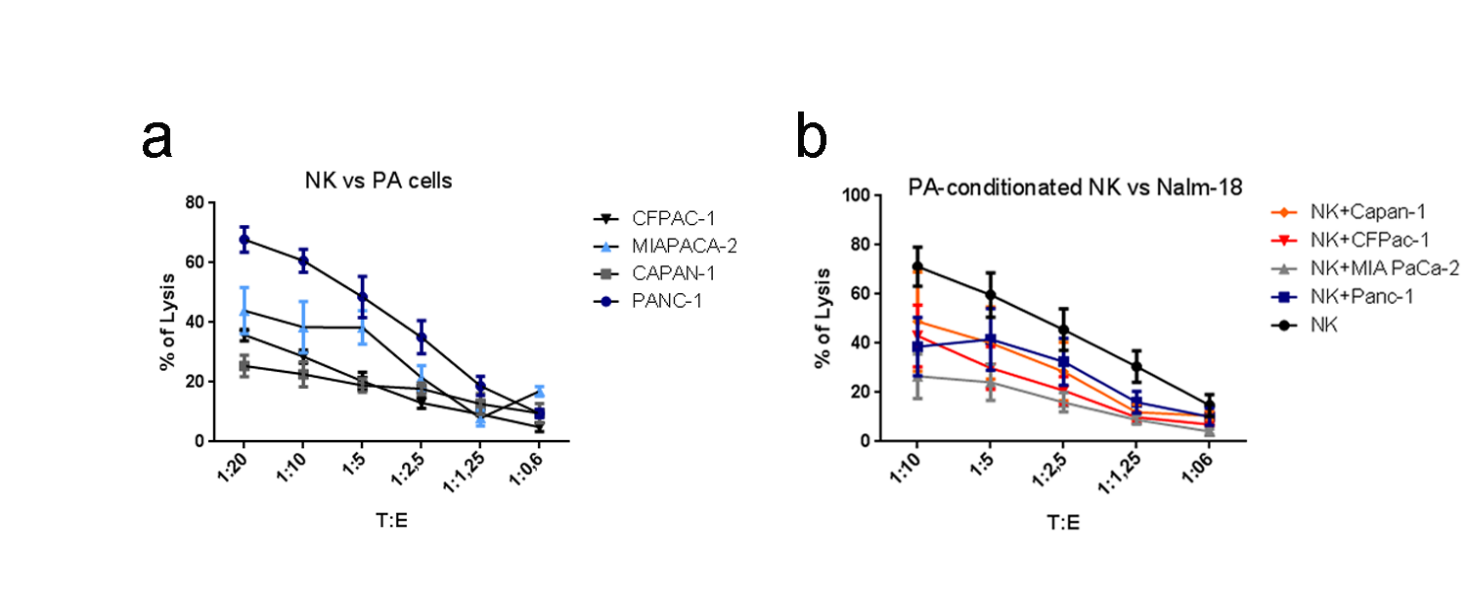


**Figure S2.** (A) Cytotoxicity of activated NK cells against metastatic PA cell lines CAPAN-1 and CFPAC-1, and non metastatic PA cell lines PANC-1 and MIAPACA-2 after 4 hours of incubation at the indicated T:E ratio. Dead cells were evaluated as PI positive cells by flow-cytometry Data of 4 independent experiments for each cell line ± SEM. (B) NK were cultured for 6 days in absence or in presence of PANC-1, CAPAN-1, MIAPACA-2 and CFPAC-1. After 6 days of co-culture, cytotoxicity assay were performed against NALM-18 at the indicated T:E ratio. Dead cells were evaluated as Propidium iodide (PI) positive cells by flow-cytometry after 4 hours. Data indicate the average of 5 independent experiments ± SEM.


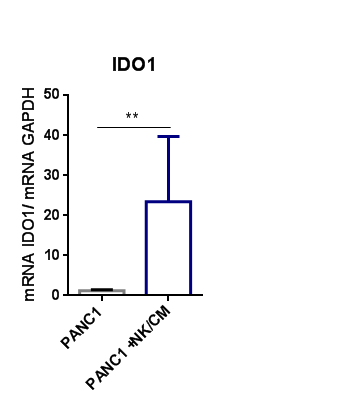


**Figure S3**. TGFβ and IDO1 expression measured by qRT-PCR in PANC-1 cell after 24 hours of incubation with NK/CM. Data of 4 independent experiments are expressed as 2-ΔCt with respect to GAPDH expression, used as endogenous control.


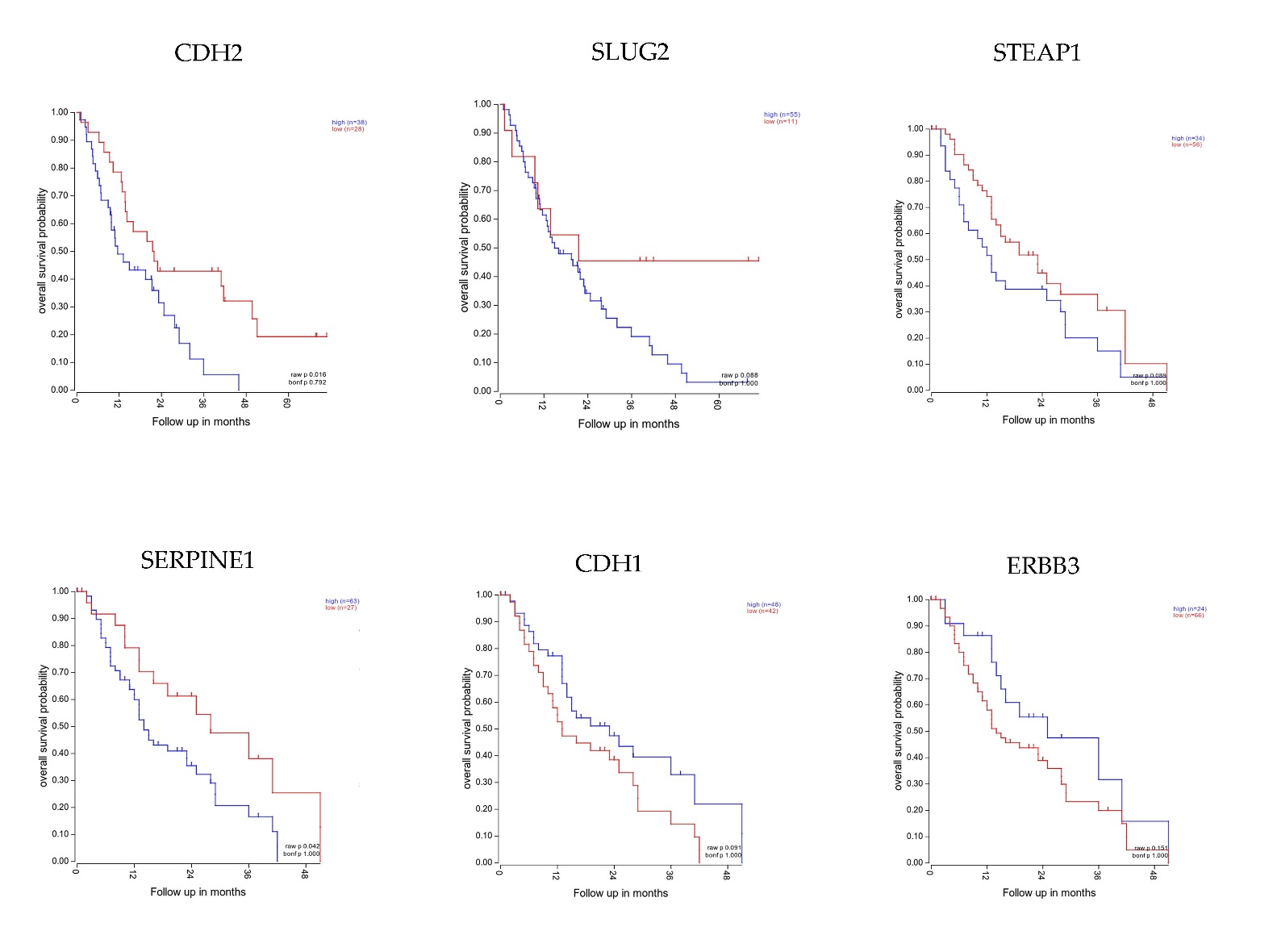


**Figure S4.** Overall survival of patients with PDAC based on the expression of mesenchymal (CDH2, SLUG2, STEAP1, SERPINE1) and epithelial (CDH1 and ERBB3) markers. The analysis was performed on R2 public dataset (Tumor pancreas Zhang dataset).


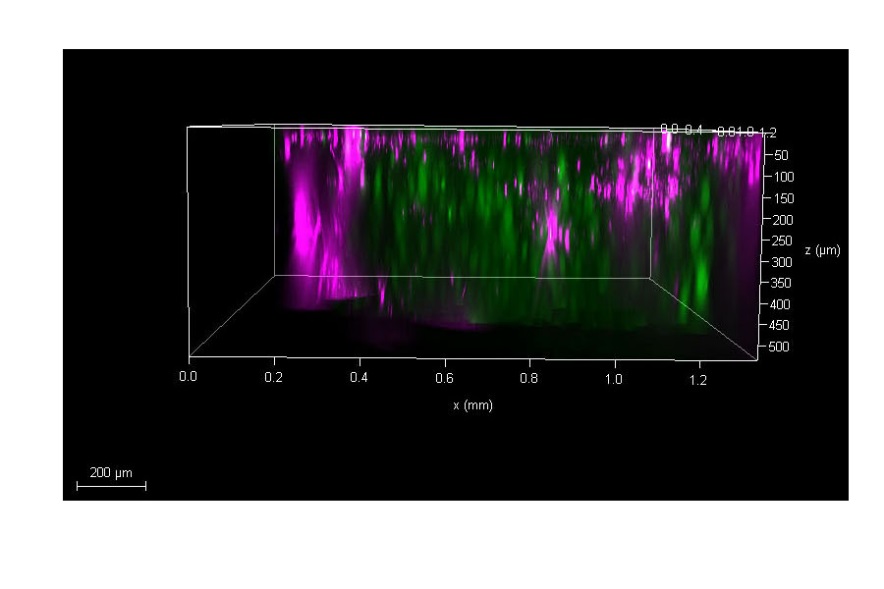


**Figure S5.** 3D microscopy model constructed from Z-stack slide of GFP-PANC-1 alginate spheres after 24 hours of co-culture with NK cells labeled in red with CM-DIL.


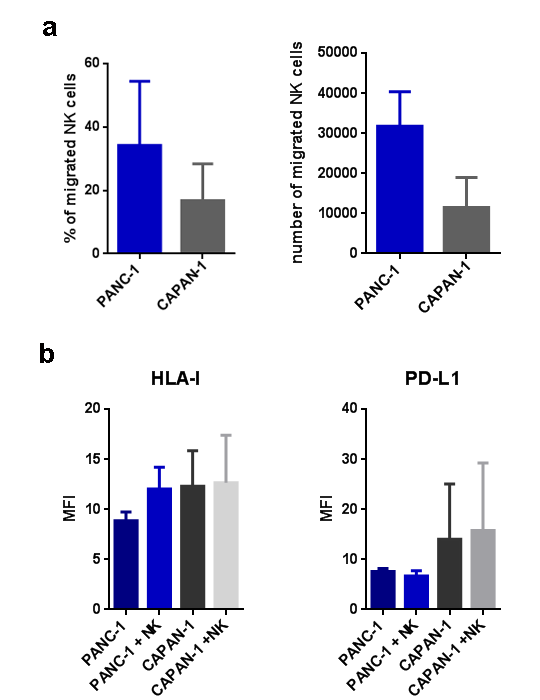


**Figure S6** (A) Percentages and absolute numbers of NK cells migrate in PANC-1 and CAPAN-1 spheroids. Data indicate the average of 3 independent experiments ± SEM. (B) HLA-I and PDL-1 expression in spheroids in the presence or in the absence of NK cells. Data indicate the average of 3 independent experiments ± SEM.
